# Imputation method to reduce undetected severe acute respiratory infection cases during the coronavirus disease outbreak in Brazil

**DOI:** 10.1590/0037-8682-0528-2020

**Published:** 2020-09-14

**Authors:** Silvano Barbosa de Oliveira, Fabiana Ganem, Wildo Navegantes de Araújo, Jordi Casabona, Mauro Niskier Sanchez, Julio Croda

**Affiliations:** 1 Universidade de Brasília, Brasília, DF, Brasil.; 2 Universidad Autónoma de Barcelona, Spain.; 3 Centre d'Estudis Epidemiològics sobre les ITS i SIDA de Catalunya (CEEISCAT), Institut Català d’Oncologia, Campus de Can Ruti, Catalunya, Spain.; 4 Centro de Investigación Biomédica en Red de Epidemiología y Salud Pública (CIBERESP), Madrid, Spain.; 5 Universidade Federal do Mato Grosso do Sul, Faculdade de Medicina, Campo Grande, MS, Brasil.; 6 Yale University School of Public Health, Department of Epidemiology of Microbial Diseases, New Haven, United States of America.; 7 Fundação Oswaldo Cruz, Campo Grande, MS, Brasil.

**Keywords:** COVID-19, SARI, Laboratory test, Signs and symptoms, Imputation method

## Abstract

**INTRODUCTION:**

The coronavirus disease (COVD-19) outbreak has overburdened the surveillance of severe acute respiratory infections (SARIs), including the laboratory network. This study was aimed at correcting the absence of laboratory results of reported SARI deaths.

**METHODS:**

The imputation method was applied for SARI deaths without laboratory information using clinico-epidemiological characteristics.

**RESULTS:**

Of 84,449 SARI deaths, 51% were confirmed with COVID-19 while 3% with other viral respiratory diseases. After the imputation method, 95% of deaths were reclassified as COVID-19 while 5% as other viral respiratory diseases.

**CONCLUSIONS:**

The imputation method was a useful and robust solution (sensitivity and positive predictive value of 98%) for missing values through clinical & epidemiological characteristics.

The coronavirus disease (COVID-19) pandemic had caused more than 10 million cases and 500,000 deaths worldwide by June 2020^1^. The severe acute respiratory syndrome coronavirus 2 (SARS-CoV-2) virus has been spreading fast globally, causing many severe cases and deaths. This virus has a higher basic reproduction number (R0) and case fatality rate (CFR) compared to influenza (R0: 2.5-3.3 and CFR: 0.4%-2.9% versus R0: 1.2-2.3 e CFR: 0.15%-0.25%, respectively)^2^ ^-^ ^5^. In Brazil, the first confirmed case was reported on February 25 in Sao Paulo City, and recently at least one case has been reported in all Brazilian states and almost all municipalities (96%)^6^.

Brazil has a surveillance system working at three levels (federal, state, and municipality) of government installed in public and private health units for severe acute respiratory illness (SARI), and notification of SARI has been mandatory since 2009. The reported cases included patients hospitalized because of SARI at any health service and mild respiratory cases reported by sentinel networks using an online database (Influenza Epidemiological Surveillance Information System in Brazil - SIVEP-GRIPE). The discovery of SARS-CoV-2 in China and suspected cases in Brazil were reported using the REDCap platform, remaining until the country reached 1,000 confirmed cases; subsequently, a new system was developed (e-SUS) and used to report mild respiratory cases, and the SARI remained reported on SIVEP. Because of the continuity and consistency, SIVEP has been maintained as an official system to report and monitor the severe cases of COVID-19, including the deaths from COVID-19 independent of hospitalization.

Although SIVEP is an online platform, inconsistencies in monitoring and case closure opportunities persist. In addition, the Ministry of Health has reported a high percentage of deaths from SARI without a diagnosis, called “non-specified SARI,” or alerted health authorities to a possible activity of other respiratory viruses in the Brazilian population. Therefore, this study was aimed at investigating the clinico-epidemiological characteristics of deaths from SARI reported in the Influenza Epidemiological Surveillance Information System in Brazil (SIVEP-Gripe) to correct the absence of robust laboratory results for COVID-19.

We used deaths from SARI reported in the Influenza Epidemiological Surveillance Information System in Brazil (SIVEP-Gripe) during the COVID-19 outbreak from January 1 to June 28, 2020. The death registers were selected using the case evolution variable.

All reported cases were classified as follows: (i) COVID-19, with laboratory confirmation through the reverse-transcriptase polymerase chain reaction (RT-PCR) for SARS-CoV-2; (ii) undetected, with laboratory confirmation through RT-PCR for other viruses; and (iii) missing value, with no confirmation through RT-PCR and an indeterminate result in the processing test. This was considered our response variable to the regression model and subsequently imputed.

Before completing the data imputation method, we performed the logistic regression analysis to identify the variables related to the response. First, we applied the univariate model using the following predictors: signs and symptoms (fever, cough, throat pain, dyspnea, respiratory distress, O_2_ saturation < 95%, diarrhea, and vomiting), comorbidities (chronic cardiovascular disease, chronic hematological disease, chronic liver disease, asthma, diabetes mellitus, chronic neurological disease, other chronic pneumopathy, immunodeficiency/immunodepression, chronic kidney disease, and obesity), hospitalization (yes/no), intensive care unit stay (yes/no), ventilation support (invasive, non-invasive, and none), chest X-ray, sex, and age group (<10 years, 10 to 39 years, 40 to 59 years, 60 to 69 years, and 70 years or more). The multiple logistic regression model was obtained from variables with a *p*-value less than 10% in the univariate regression model, and stepwise method was applied using the Akaike information criterion, Bayesian information criterion, and deviance. Subsequently, cases classified as “missing value” were subjected to a data imputation method using as predictors the variables selected in the multiple logistic regression.

We applied the multiple imputation method to obtain complete information for the “missing value” cases for the classification of SARI deaths. Imputation was performed using the additive regression method, which comprised procedures of a flexible additive model (nonparametric regression method) fitted on samples taken with replacements from original data and missing values (dependent variable) and predicted using non-missing values (independent variable obtained by multiple logistic regression)^7^ ^-^ ^9^.

We selected a random sample of SARI deaths that had resulted from COVID-19 and other viral respiratory diseases to validate the data imputation method. It generated randomly missing values for 30% of cases, and we applied the imputation method. Subsequently, the imputed values were compared with the observed values. The sensitivity, specificity, positive predictive value, and negative predictive value were calculated to quantify this validation. Furthermore, the Kappa test was performed to measure the concordance between the imputed and observed values. The significance level was considered as 5% for all analyses. All data were processed using R software, and the data imputation method was performed using the R package Hmisc.

In Brazil, from January 1 to July 28, 2020, 84,449 deaths from SARI were reported. Furthermore, 45,321 (54%) cases were confirmed using RT-PCR for some respiratory viruses, of which 42,981 (95%) were confirmed as COVID-19. These proportions of confirmed COVID-19 cases were different across Brazilian states, with the lowest in the Mato Grosso do Sul (19%) state and the highest in Acre (91%) (Table 1).

Considering the overall deaths reported in Brazil, the number of cases undetected for respiratory viruses, indeterminate in RT-PCR, not tested, in processing, and without information were 21,770 (26%), 553 (1%), 2,829 (3%), 6,404 (8%), and 7,571 (9%), respectively; all of these cases were considered as “missing value,” totaling 39,128 (46%) registers. Important variations were also observed across Brazilian states, highlighting the following five states with the highest proportions of missing value: Minas Gerais (76%), Mato Grosso do Sul (75%), Rio Grande do Sul (73%), Paraná (73%), and Santa Catarina (69%) (Table 1).

**TABLE 1:** Death from severe acute respiratory illness classified by laboratory results reported by Brazilian states (Brazil, January to July, 2020)

UF	Total	COVID-19		Other viral respiratory diseases		Missing*	
	nº	nº	%	nº	%	nº	%
Brazil	84449	42981	51	2340	3	39128	46
AC	203	184	91	4	2	15	7
AL	1234	618	50	55	4	561	45
AM	4144	1829	44	80	2	2235	54
AP	191	94	49	0	0	97	51
BA	2824	1472	52	176	6	1176	42
CE	7579	3683	49	302	4	3594	47
DF	902	486	54	17	2	399	44
ES	1321	880	67	70	5	371	28
GO	940	401	43	34	4	505	54
MA	2530	944	37	308	12	1278	51
MG	3133	688	22	75	2	2370	76
MS	345	66	19	19	6	260	75
MT	311	99	32	29	9	183	59
PA	5825	3124	54	199	3	2502	43
PB	1470	557	38	93	6	820	56
PE	5243	3834	73	65	1	1344	26
PI	665	346	52	33	5	286	43
PR	2169	530	24	59	3	1580	73
RJ	13019	7514	58	225	2	5280	41
RN	1005	509	51	36	4	460	46
RO	247	135	55	13	5	99	40
RR	337	134	40	3	1	200	59
RS	2009	530	26	6	0	1473	73
SC	811	243	30	12	1	556	69
SE	302	168	56	18	6	116	38
SP	25411	13754	54	406	2	11251	44
TO	265	151	57	3	1	111	42

Source: SIVEP-GRIPE accessed in 06/20/2020. Notes: *included undetected results, indeterminate, not tested, in processing, ignored, and missing.

In the univariate logistic regression model, the age group was associated with COVID-19 and positively correlated with the odds ratio. The signs and symptoms that showed significant associations were respiratory distress, fever, cough, throat pain, and dyspnea, all indicating inverse odds to be detected for COVID-19. Only four underlying health conditions presented with significant associations with COVID-19: chronic cardiovascular disease, diabetes mellitus, chronic kidney disease, and obesity. Individuals that needed intensive care were more likely to be detected with COVID-19. In the multiple logistic regression, only five variables remained in the final model: age group, with age of 40 years or above having approximately eight times more odds to be detected with COVID-19 compared to age below 10 years; 33% chance for individuals with respiratory distress; 10% to 20% more chance for individuals with chronic cardiovascular disease and diabetes mellitus, respectively; and increasing chance in individuals who require ventilation support (32%: invasive; 38%: non-invasive) (Table 2).

**TABLE 2:** Demographic information and logistic regression for death from severe acute respiratory illness confirmed to be coronavirus disease in the reverse-transcriptase polymerase chain reaction test (Brazil, January to July, 2020).

Variables	Total	COVID-19		Univariate	Multivariate
	nº	nº	%	OR (95% CI)	OR (95% CI)
**Total**	45321	42981	94.8	-	-
Sex					
Male	26378	24993	94.7	1.00	-
Female	18929	17977	95.0	1.05 (0.96-1.14)	-
Missing	14	11	78.6	-	-
Age group					
<10 years	224	161	71.9	1.00	1.00
10 to 39 years	2318	2141	92.4	4.73 (3.41-6.58)	5.72 (3.08-10.39)
40 to 59 years	9708	9208	94.8	7.21 (5.31-9.78)	7.71 (4.32-13.34)
60 to 69 years	10341	9826	95.0	7.47 (5.51-10.12)	8.51 (4.76-14.77)
>70 years	22730	21645	95.2	7.81 (5.80-10.51)	8.14 (4.60-13.94)
Signs and symptoms					
Respiratory distress	28634	26942	94.1	0.69 (0.61-0.78)	0.77 (0.65-0.92)
Fever	29889	28251	94.5	0.82 (0.73-0.92)	-
Cough	31431	29765	94.7	0.87 (0.77-0.99)	-
Throat pain	7362	6908	93.8	0.80 (0.72-0.90)	-
Dyspnea	34513	32618	94.5	0.86 (0.75-0.98)	-
O_2_ saturation <95%	29416	27821	94.6	0.95 (0.85-1.07)	-
Diarrhea	4688	4453	95.0	1.03 (0.89-1.19)	-
Vomit	2770	2606	94.1	0.86 (0.72-1.01)	-
Comorbidities					
Chronic cardiovascular disease	17682	16926	95.7	1.25 (1.10-1.41)	1.10 (0.95-1.28)
Diabetes mellitus	14037	13397	95.4	1.15 (1.02-1.30)	1.20 (1.04-1.40)
Chronic hematological disease	521	496	95.2	1.04 (0.69-1.56)	-
Chronic liver disease	590	567	96.1	1.29 (0.84-1.97)	-
Asthma	935	880	94.1	0.83 (0.62-1.10)	-
Chronic neurological disease	2698	2572	95.3	1.05 (0.86-1.27)	-
Other chronic pneumopathy	2384	2267	95.1	1.02 (0.84-1.25)	-
Immunodeficiency/immunodepression	1744	1662	95.3	1.05 (0.83-1.33)	-
Chronic kidney disease	2926	2811	96.1	1.30 (1.06-1.59)	-
Obesity	1700	1639	96.4	1.45 (1.11-1.89)	-
Hospitalization					
No	2168	2065	95.2	1.00	-
Yes	40429	38466	95.1	0.98 (0.80-1.20)	-
Missing	4892	4515	92.3	-	-
Intensive care unit stay					
No	14289	13525	94.7	1.00	-
Yes	20426	19464	95.3	1.14 (1.04-1.26)	-
Missing	24895	23517	94.5	-	-
Ventilation support					
No	5131	4839	94.3	1.00	1.00
Yes, invasive	16944	16061	94.8	1.10 (0.96-1.26)	1.32 (1.08-1.61)
Yes, non-invasive	12065	11516	95.4	1.27 (1.09-1.47)	1.38 (1.11-1.70)

Source: SIVEP-GRIPE accessed in 06/20/2020

Using the variables defined by the multiple logistic regression, the imputation method was applied for all data classified as “missing value.” Of the total registers classified as “missing value,” the data imputation method could classify 37,980 cases (97%). Furthermore, 1,994 (2%) cases were detected with other viral respiratory diseases (undetected for COVID-19), and 35,986 (43%) cases were confirmed with COVID-19. Therefore, of the total deaths from SARI that occurred in Brazil from January 1 to July 28, 2020, 95% were reclassified as COVID-19 while 5% as some other viral respiratory disease (not COVID-19). Hence, all Brazilian states and federal district have at least 90% of deaths from SARI classified as COVID-19. Only the Maranhão (15%), Mato Grosso (14%), and Mato Grosso do Sul (11%) states presented with more than 10% of SARI deaths classified as other viral respiratory diseases by the imputation data method (Table 3).

**TABLE 3:** Imputed classification of death from severe acute respiratory illness by Brazilian states (Brazil, January to July, 2020).

UF	Total	COVID-19		Other viral respiratory	
	nº	nº	%	nº	%
Brazil	84449	80022	95	4427	5
AC	203	198	98	5	2
AL	1234	1134	92	100	8
AM	4144	3956	95	188	5
AP	191	187	98	4	2
BA	2824	2579	91	245	9
CE	7579	7082	93	497	7
DF	902	869	96	33	4
ES	1321	1233	93	88	7
GO	940	878	93	62	7
MA	2530	2147	85	383	15
MG	3133	2944	94	189	6
MS	345	307	89	38	11
MT	311	267	86	44	14
PA	5825	5494	94	331	6
PB	1470	1334	91	136	9
PE	5243	5103	97	140	3
PI	665	614	92	51	8
PR	2169	2022	93	147	7
RJ	13019	12512	96	507	4
RN	1005	948	94	57	6
RO	247	231	94	16	6
RR	337	326	97	11	3
RS	2009	1924	96	85	4
SC	811	770	95	41	5
SE	302	281	93	21	7
SP	25411	24409	96	1002	4
TO	265	260	98	5	2

Source: SIVEP-GRIPE accessed in 06/20/2020.

To validate the data imputation method, simulation showed high sensitivity (99%) and positive predictive value (99%) and substantial values of specificity (71%) and negative predictive value (73%). Moreover, the Kappa test showed substantial concordance between the imputation method and the observed SARI reported (K= 0.71; *p*-value < 0.001) (Supplementary Table 1).

The absence of information in the test causing undetected cases of viral respiratory diseases is a bias in the information system and understanding the spreading of COVID-19 or other viral respiratory diseases in Brazil because only information about detected tests is reported in SIVEP-Gripe. Almost half of SARI deaths have an unknown cause; 26% of SARI deaths had undetected RT-PCR results; however, we do not know which respiratory viruses were tested, and more than 20% of deaths were not tested.

The simulation of the data imputation method from the real values proved a useful and robust solution to resolve the problem of the missing values or undetected results without identifying which respiratory viruses were tested using the clinical & epidemiological variables. This method presented a high sensitivity and positive predictive value and substantial values of specificity and negative predictive value, such as a moderate concordance with the real value using the simulation. Another way to validate this method is selecting some imputed cases and trying to investigate the medical records to identify more examinations (X-ray, tomography, etc.) that help confirm the cases and perform retesting for these cases using a different methodology suitable for laboratory collection. These estimations should be confirmed with empirical data as the quality of the information systems improve.

The main limitation of this method is the associated data structure, i.e., if the quality of information is not reasonably good, the output of imputation follows this bias. With the speed of disease spread in the country, surveillance may compromise the quality of filling out epidemiological antecedents. This can explain the difference observed in some states that showed less than 90% of detected COVID-19 cases. These states usually have worse filling of the investigation form (Maranhão missing value for variables ranging from 12% to 74% while Mato Grosso and Mato Grosso do Sul ranging from 3% to 67%).
