# Supplementary figures and images for: Imputation method to reduce undetected severe acute respiratory infection cases during the coronavirus disease outbreak in Brazil
Source: Rev Soc Bras Med Trop. 2020 Sep 14;53:e20200528. doi: 10.1590/0037-8682-0528-2020 (PMC7491560; doi:10.1590/0037-8682-0528-2020)

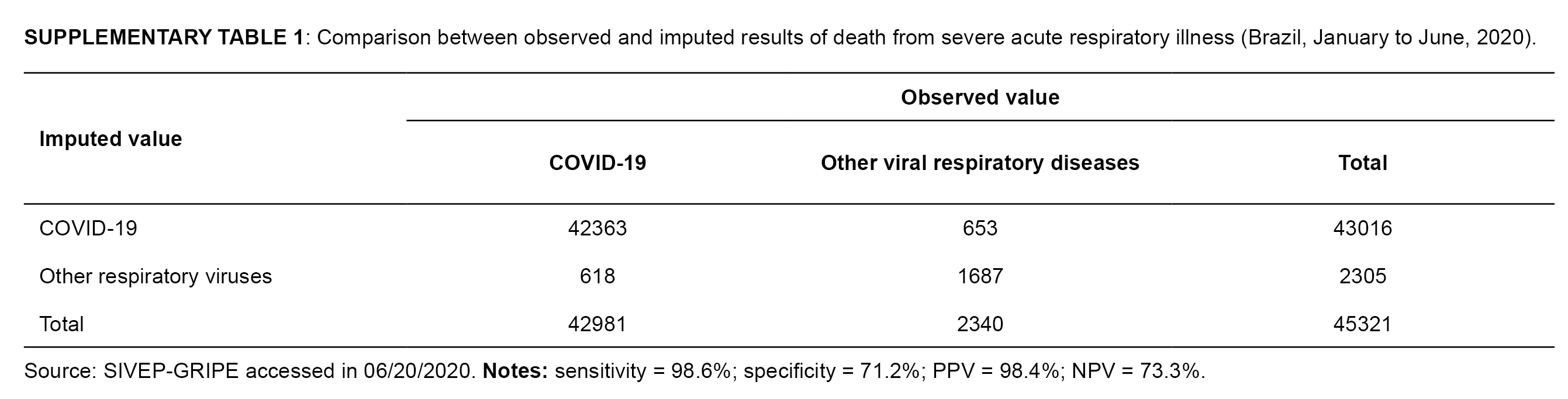

Supplement: Supplementary file 1 [file 1678-9849-rsbmt-53-e20200528-suppl1.jpg]
